# Transforaminal Lumbar Interbody Fusion with Rigid Interspinous Process Fixation: A Learning Curve Analysis of a Surgeon Team’s First 74 Cases

**DOI:** 10.7759/cureus.1290

**Published:** 2017-05-30

**Authors:** Patrick Doherty, Arthur Welch, Jason Tharpe, Camille Moore, Chris Ferry

**Affiliations:** 1 Neurosurgery, Lawrence + Memorial Hospital; 2 Division of Biostatistics and Bioinformatics, National Jewish Health; 3 Research and Development, Zimmer Biomet Spine

**Keywords:** transforaminal lumbar interbody fusion, interspinous process fixation, tlif, ispf, mis, learning curve, degenerative spine, spine, minimally invasive, pedicle screw fixation

## Abstract

**Background:**

Studies have shown that a significant learning curve may be associated with adopting minimally invasive transforaminal lumbar interbody fusion (MIS TLIF) with bilateral pedicle screw fixation (BPSF). Accordingly, several hybrid TLIF techniques have been proposed as surrogates to the accepted BPSF technique, asserting that less/fewer fixation(s) or less disruptive fixation may decrease the learning curve while still maintaining the minimally disruptive benefits. TLIF with interspinous process fixation (ISPF) is one such surrogate procedure. However, despite perceived ease of adaptability given the favorable proximity of the spinous processes, no evidence exists demonstrating whether or not the technique may possess its own inherent learning curve. The purpose of this study was to determine whether an intraoperative learning curve for one- and two-level TLIF + ISPF may exist for a single lead surgeon.

**Methods:**

Seventy-four consecutive patients who received one- or two-Level TLIF with rigid ISPF by a single lead surgeon were retrospectively reviewed. It was the first TLIF + ISPF case series for the lead surgeon. Intraoperative blood loss (EBL), hospitalization length-of-stay (LOS), fluoroscopy time, and postoperative complications were collected. EBL, LOS, and fluoroscopy time were modeled as a function of case number using multiple linear regression methods. A change point was included in each model to allow the trajectory of the outcomes to change during the duration of the case series. These change points were determined using profile likelihood methods. Models were fit using the maximum likelihood estimates for the change points. Age, sex, body mass index (BMI), and the number of treated levels were included as covariates.

**Results:**

EBL, LOS, and fluoroscopy time did not significantly differ by age, sex, or BMI (p ≥ 0.12). Only EBL differed significantly by the number of levels (p = 0.026). The case number was not a significant predictor of EBL, LOS, or fluoroscopy time (p ≥ 0.21). At the time of data collection (mean time from surgery: 13.3 months), six patients had undergone revision due to interbody migration. No ISPF device complications were observed.

**Conclusions:**

Study outcomes support the ideal that TLIF + ISPF can be a readily adopted procedure without a significant intraoperative learning curve. However, the authors emphasize that further assessment of long-term healing outcomes is essential in fully characterizing both the efficacy and the indication learning curve for the TLIF + ISPF technique.

## Introduction

As spine surgery continues to shift towards a ‘less’ or ‘minimally’ invasive (MIS) driven model, the issues of procedural feasibility and surgeon adaptability remain of particular scrutiny. While MIS techniques can succeed in diminishing tissue trauma, the prospect for complication and/or less favorable outcomes during surgeon adoption is not insignificant. These trends have been particularly pronounced within the MIS transforaminal lumbar interbody fusion (TLIF) + bilateral pedicle screw fixation (BPSF) platform, with multiple reports demonstrating a procedural learning curve [1-5].

Accordingly, there has been a push to identify alternative or hybrid MIS TLIF techniques in which adoption can be achieved more readily. These hybrid techniques often leverage less posterior fixation (i.e., unilateral pedicle screw fixation (UPSF) vs. BPSF) and/or less disruptive fixation (i.e., USPF + contralateral facet screw fixation (FSF) vs. BPSF); postulating that decrease in operative requirement may marginalize any associated learning curve [6-11].

However, despite the perceived adoption benefits of these hybrid techniques, little evidence exists definitively showing whether or not they possess their own inherent learning curve. While many employ technical components that may be considered ‘commonplace’ or ‘standard’, consideration must still be given to the fact that surgeons are often adopting these techniques while actively practicing, without opportunity for extensive training. Additionally, in some cases, no predicate (i.e., open or mini-open) or parent technique exists from which prior anatomical or technical familiarity can be expanded. For example, in the case of TLIF + BPSF, previous exposure to the open procedure provides a baseline understanding upon which the MIS approach can be furthered. However, some hybrid techniques, such as TLIF with rigid interspinous process fixation (ISPF), have no predicate procedure for which the end-stage instrumentation remains the same.

Furthermore, current/previous use of similar or comparable techniques/technologies may skew a surgeon’s ability to readily optimize a next-generation modality. Rigid ISPF is an example of this when considering the various commercially available interspinous spacers/distraction devices [12]. Despite anatomical access being near identical between technologies, ISPF utilizes rigid fixation to support fusion while interspinous spacers typically provide segmental distraction in the absence of fusion or additional instrumentation. These differences in mechanism-of-action can make early adoption more challenging, as the surgeon must address previous tendencies and acclimate to altered biomechanical nuances.

**Figure 1:**
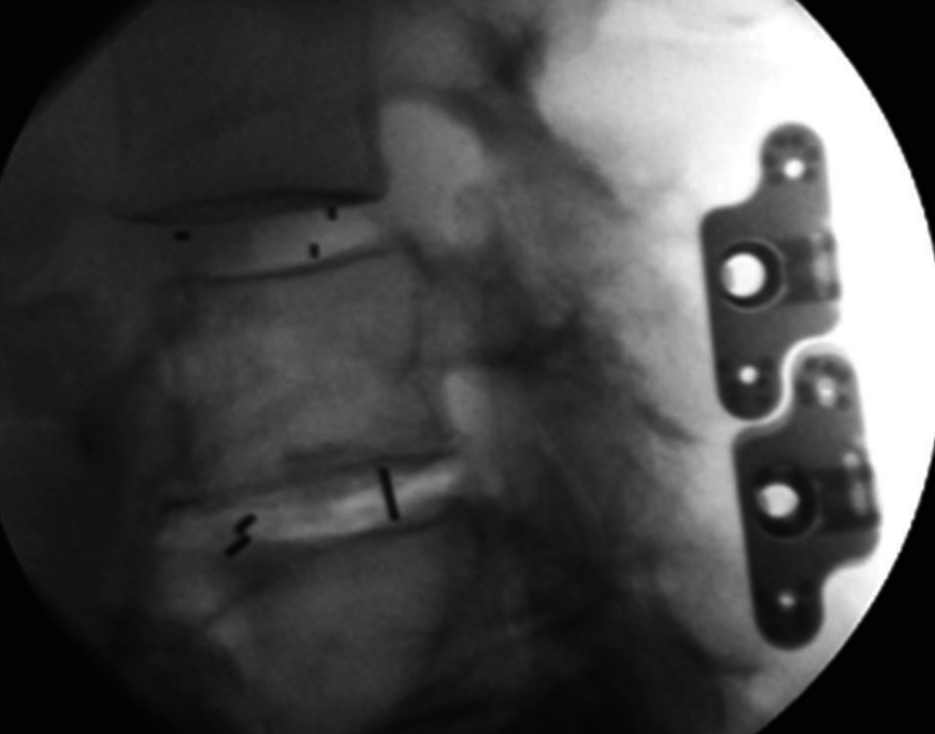
Two-level transforaminal lumbar interbody fusion with interspinous process fixation; intraoperative fluoroscopic image (lateral view)

**Figure 2:**
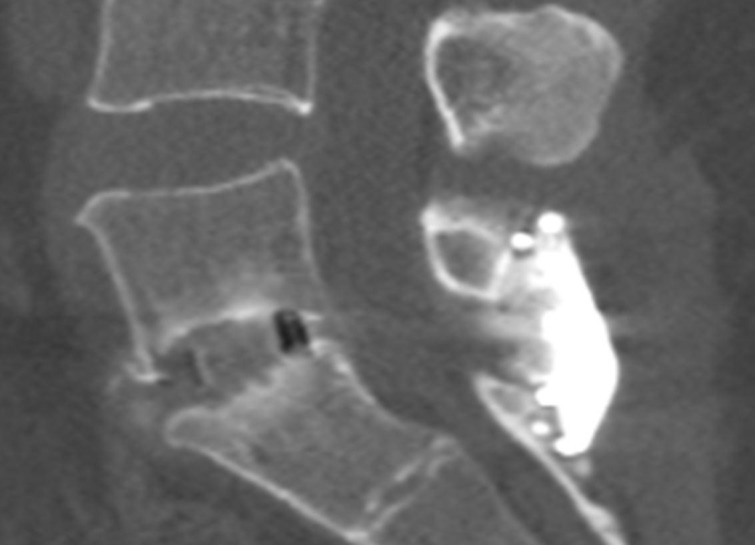
One-level transforaminal lumbar interbody fusion with interspinous process fixation; post-operative computed tomography scan (lateral view)

The purpose of this study was to assess whether there could be an intraoperative learning curve associated with TLIF + ISPF (one- and two-level) (Figures 1-2). ISPF, which was first introduced in 2006, has been widely perceived as a minimally disruptive adjunct to interbody fusion given its favorable posterior proximity; however, limited evidence exists characterizing the technique [13]. While the authors believe that the favorable access of ISPF may mitigate the challenges traditionally seen with the adoption of MIS fixation techniques, the potential for spinous process fracturing, device migration, and dural tears are not insignificant [13]. Hence, consideration of procedural feasibility and associated complication is necessary in fully characterizing the efficacy of ISPF in TLIF.

## Materials and methods

### Patient population and study design

Seventy-four patients underwent one- or two-level TLIF with supplemental ISPF at the same practice. All procedures were performed by the same surgical team under the guidance of the same senior lead surgeon (PD). This was the first case series performing TLIF + ISPF for this team. All cases were performed within a span of 524 working days; during which time all patients considered candidates for traditional TLIF + BPSF (one- or two-level) received the hybrid TLIF + ISPF technique. Contraindications for ISPF use were: 1) compromised spinous processes and/or 2) presence of an isthmic spondylolisthesis.

Patient charts were retrospectively reviewed. Institutional review board approval was obtained prior to data collection (Western IRB, Puyallup, WA, USA). Analysis inclusion criteria required patients to have undergone TLIF with ISPF (only), at all treated levels, for degenerative changes in the lumbar spine. Primary diagnoses included degenerative disc disease (DDD), herniated nucleus pulpous (HNP), lumbar instability, recurrent HNP, spondylolisthesis, and stenosis. Patients with prior fusion surgery at the index level(s) were excluded. All diagnoses were confirmed by plain anterioposterior (A/P) and lateral radiographs, computed tomography (CT) scans, and/or magnetic resonance imaging (MRI). Six months of conservative treatment had been either non-responsive or insufficient.

### Statistical methods

Patient covariate data for age, sex, body mass index (BMI), primary, secondary, and tertiary diagnoses, and prior surgeries was collected. Estimated intraoperative blood loss (EBL), patient length of hospital stay (LOS), intraoperative fluoroscopy time, and perioperative complications were recorded and stratified by age (<60 and ≥60 years), BMI (<30 kg/m^2^ and ≥30 kg/m^2^), and number of surgical levels (one- and two-level).

To determine if there was an intraoperative learning curve, EBL, LOS and fluoroscopy time were modeled as a function of case number using multiple linear regression (SAS version 9.0, PROC REG). A change point was included in each model to allow the trajectory of the outcomes to change during the duration of the case series. These change points were determined using profile likelihood methods described by Hall, et al. [14]. A sequence of change points from one to 74 cases was considered and models were fit using the maximum likelihood estimates for the change points. In addition, potential confounders were included as covariates in the models. These included: sex, age, BMI, and the number of fusion levels. A final model was chosen using backward selection. Standard errors were estimated using a non-parametric bootstrap to account for the additional uncertainty introduced by estimating the change point. A significance level of 0.05 was used for all statistical tests.

### Learning curve metric rationale

EBL, LOS, and fluoroscopy time were specifically chosen as key indicators of an intraoperative learning curve in this analyses, as they speak to the surgeons’ ability to preserve the paraspinous environment and characterize ease of hardware placement. The authors assert that if a significant decrease in EBL and LOS occurred over the course of the case series that it would be indicative of the surgeon continuing to adapt to the intricacies of the approach. Furthermore, the authors assert that the amount of fluoroscopic imaging utilized during the procedure is indicative of the difficulty of hardware placement, with greater fluoroscopy time indicating greater difficulty in placement. If a significant decrease in fluoroscopy time occurred during the case series it would be a reflection of the surgeon becoming more comfortable with navigation and hardware placement.

Additionally, the authors acknowledge that operative times and patient reported outcomes are often used to characterize procedural learning curve [1-5]. However, these metrics were purposefully not employed in this analysis. While operative time trends have the potential to demonstrate surgeon familiarity or adaptability, they should only be considered when intraoperative and perioperative outcomes are not significantly changing. These outcomes becoming asymptotic would indicate that procedural execution or intraoperative ‘craftsmanship’ had become uniform. Unless procedural execution is uniform, operative time will not accurately characterize the degree to which surgeon adaptability is changing without subsequent changes in intraoperative invasiveness. Just because a surgeon is becoming faster at a procedure does not necessarily mean that they are becoming better at the procedure or achieving the same quality of correction. Furthermore, ergonomic factors such as scrub tech instrumentation familiarity, first assist workflow optimization, microscope manipulation, etc. can all impact operative time, particularly when introducing a new surgical system or technique. Lastly, when considering patient-reported outcomes, the question of adaptability is no longer a function of intraoperative craftsmanship alone, but also of procedural indication. A surgeon may be improving their technical surgical aptitude with each subsequent case; however, if they continue to misappropriate the procedure, then the outcomes are representative of an indication learning curve, not a technical procedural learning curve. The authors urge that the learning curves associated with intraoperative aptitude and procedural indication should be considered two unique entities. Furthermore, as with operative time, the indication learning curve should only be assessed when intraoperative aptitude becomes consistent. The purpose of this study was to only assess the intraoperative learning curve of TLIF + ISPF.

### Surgeon experience

The lead surgeon of this study is a residency trained neurosurgeon of 17 years in practice. The ISPF technique was first observed and implemented into their practice in approximately 2010, after 11 years of practicing spine surgery. PSF was the predominate means of supplemental fixation previously. Some prior exposure to interspinous modalities was acquired through the use of static interspinous spacers for spinal stenosis. However, no formal training in rigid ISPF was received prior to adopting it into practice. The rationale for adoption was the proposed intraoperative benefits of diminished fluoroscopic exposure, operative time, blood loss, and reduced potential for complications including vertebral breaches and nerve irritation/injury. Indication for ISPF was determined as any indication in which PSF was an option, as long as the spinous processes were intact and there was no evidence of an isthmic spondylolisthesis. The authors assert that the lead author, and subject of this investigation, is an appropriate representation of a surgeon in which the adoption of an alternative technique could potentially prove challenging given a lack of previous exposure and formal training.

### Surgical technique

After induction of general anesthesia, the patients were placed in the prone position on a radiolucent table. Confirmation of operative levels was performed by palpation and fluoroscopic imaging. A 3 cm midline incision was made over the spinous process(es) and the musculature was incised via a standard midline approach. The spinous processes and lamina were exposed to the medial border of the facet joints, while preserving the supraspinous ligament.

A conservative microsurgical foraminotomy was performed unilaterally on the symptomatic side of each treatment level. Bone fragments obtained during surgery were kept as graft material for subsequent packing of the device barrel, interbody (IB) implant graft cavity, intervertebral void space, facet joints, and for laminar on-lay, when deemed necessary and safe. Care was given as not to remove bone aggressively, or in an excessive manner, but to ensure the integrity of the concurrent and adjacent spinous processes needed for subsequent fixation.

In the same approach, disc material and cartilaginous endplates were removed transforaminally with reaming, curette, and forcep instrumentation. Autograft was packed anteriorly within the disc space. The IB cage(s) was then impacted into the interdiscal space, along with morselized bone fragments, supplemental demineralized bone matrix, and/or bone graft substitute when necessary. Partial facetectomies of hypertrophic facets on the ipsilateral and/or contralateral side were performed when necessary to allow for proper placement of the ISPF device. Appropriate implant positioning was confirmed via A/P and lateral fluoroscopic imaging.

Following IB cage placement and decompression, the interspinous ligament was punctured as far anteriorly as possible using a dilator. Using a spreader within the interspinous space, the appropriate size of the ISPF implant (ASPEN® MIS Fusion System, Zimmer Biomet Spine, Broomfield, CO, USA; Figure 1) was determined. The spinous processes were then decorticated using a rasp. The post-plate body of the device was placed first, anatomically to the left of the spinous processes, with the barrel portion packed with graft material of choice. In multi-level cases, the devices were placed cephalad to caudad. The matting lock plate was then placed over the plate-post on the contralateral side of the spinous processes such that intimate contact was made with bone. The device was placed as anteriorly as possible in order to access/grip the thicker bone mass at the laminar junction. It was ensured that the device did not protrude above the lumbodorsal fascia and that the fixation spikes effectively engaged the spinous processes prior to final compression. Additional angulation of the plates was performed if necessary. Final plate compression and set-screw tightening was performed. Final device placement was confirmed via A/P and lateral fluoroscopic imaging.

## Results

Patient demographic and outcome data is summarized in Tables 1-4.

**Table 1:** Patient demographics DDD: Degenerative disc disease; HNP: Herniated nucleus pulposus.

N (%)	Total	One-Level	Two-Level
Number of Subjects	74	50 (67.6%)	24 (32.4%)
Sex (% Female)	48 (64.9%)	37 (74%)	11 (45.8%)
Primary Diagnosis			
DDD	1 (1.4%)	1 (2%)	0
HNP	2 (2.7%)	2 (4%)	0
Instability	2 (2.7%)	2 (4%)	0
Recurrent HNP	2 (2.7%)	2 (4%)	0
Spondylolisthesis	13 (17.6%)	12 (24%)	1 (4.2%)
Stenosis	54 (72.9%)	31 (62%)	23 (95.8%)
Prior Surgery(s) (Non-Index)	17 (23.0%)	14 (28%)	3 (12.5%)

**Table 2:** Primary, secondary, and tertiary diagnoses DDD: Degenerative disc disease; HNP: Herniated nucleus pulposus.

Diagnosis	N	Percent (%)
Stenosis	66	89.2
DDD	62	83.8
Instability	54	73
Spondylolisthesis	23	31.1
HNP	9	12.2
Facet Cyst	1	1.4

**Table 3:** Patient demographics and perioperative outcomes by number of levels *Denotes p < 0.05, EBL: Two-Level fusion patients significantly greater than one-level patients. BMI: Body mass index; EBL: Estimated blood loss; SD: Standard deviation.

	Mean	SD	Median	Minimum	Maximum
Age (Years)	56.6	12.6	54.0	31	84
*One-Level*	55.1	12.2	53.0	31	80
*Two-Level*	59.1	13.1	62.0	33	84
BMI (kg/m^2^)	29.1	6.8	28.0	15	52
*One-Level*	28.5	6.5	27.7	18	47
*Two-Level*	30.2	7.4	28.9	15	52
Length-of-Stay (days)	3.0	1.3	3.0	1	6
*One-Level*	2.9	1.3	3.0	1	6
*Two-Level*	3.2	1.4	3.0	1	6
EBL (ml)	261.6	293.4	175.0	25	1500
*One-Level*	184.8	189.0	100.0	25	1100
*Two-Level*	395.4*	386.3	275.0	50	1500
Fluoroscopy Time (sec)	35.4	26.8	30.0	6	156
*One-Level*	31.4	26.1	24.0	10	156
*Two-Level*	42.1	27.2	33.0	6	132

**Table 4:** Perioperative outcomes stratified by patient age and body mass index *Note: Eight (n = 8) subjects had insufficient demographic data to calculate BMI. BMI: Body mass index; SD: Standard deviation.

	Age (years)		BMI (kg/m^2^)	
	< 60	≥ 60	< 30	≥ 30
N (%)	41 (55.4%)	33 (44.6%)	41 (62.1%)*	25 (37.9%)*
Length-of-stay (days)				
Mean	2.8	3.4	3.1	3
SD	1.2	1.4	1.5	1.3
Median	3	3	3	3
Minimum	1	1	1	1
Maximum	6	6	6	6
EBL (ml)				
Mean	257.6	266.7	212.4	312
SD	329	246.9	204.6	342.6
Median	100	200	125	200
Minimum	25	50	25	50
Maximum	1500	1400	1100	1400
Intraoperative Fluoroscopy Time (sec)				
Mean	32.5	39.4	36	38.9
SD	18.9	34.6	25.5	31.2
Median	24	30	30	29.5
Minimum	10	6	12	6
Maximum	78	156	156	132

### Intraoperative estimated blood loss

**Figure 3:**
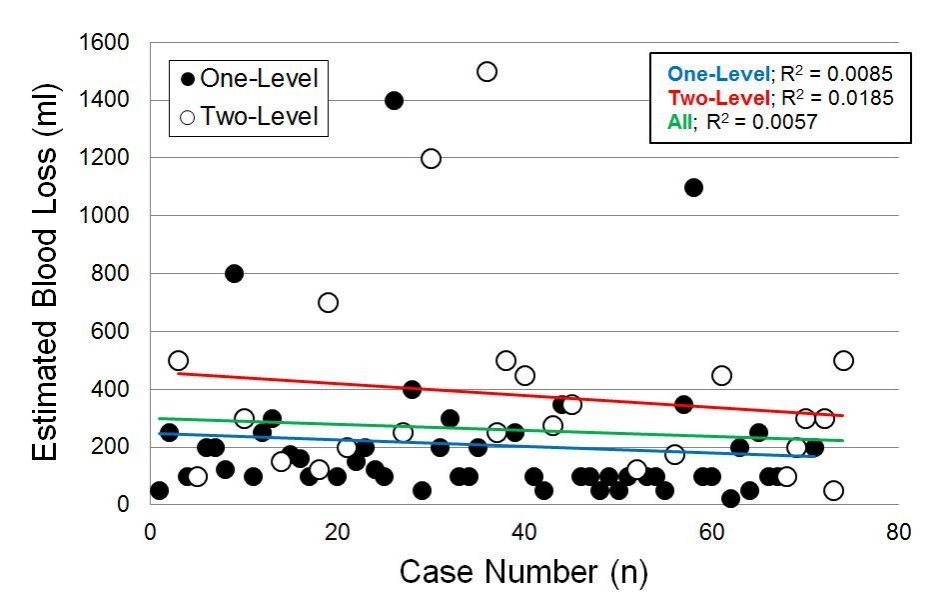
Intraoperative blood loss vs. case number; case number was not a significant predictor (p = 0.22)

Intraoperative EBL did not significantly differ by age, sex, or BMI (p ≥ 0.66). Only the total number of surgical levels was significantly associated with EBL (p = 0.026) (Tables 3-4). When modeling EBL as a function of the case number, the most likely change point was after 32 cases. However, in the multiple linear regression model, the case number was not a significant predictor of EBL (p = 0.22) and there was no significant difference in EBL before and after the 32nd case (p = 0.10) (Figure 3).

### Intraoperative fluoroscopy time

**Figure 4:**
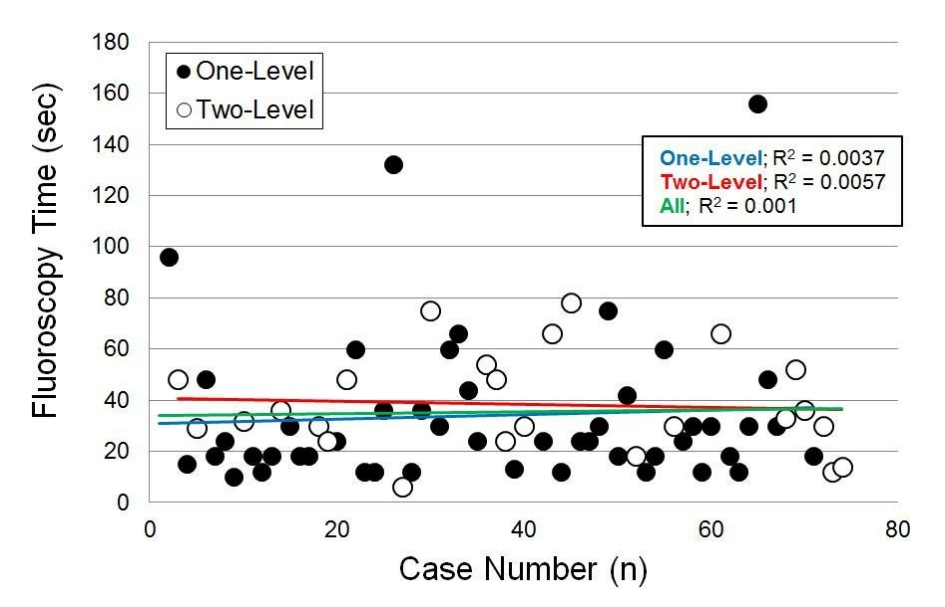
Intraoperative fluoroscopy time vs. case number; case number was not a significant predictor (p = 0.38)

Intraoperative fluoroscopy time did not significantly differ by age, sex, the number of surgical levels, or BMI (p ≥ 0.12) (Tables 3-4). When modeling fluoroscopy time as a function of the case number, the most likely change point was after 33 cases. However, in the multiple linear regression model, the case number was not a significant predictor of fluoroscopy time (p = 0.38) and there was no significant difference between fluoroscopy time before and after the 33rd case (p = 0.66) (Figure 4).

### Hospitalization length-of-stay

**Figure 5:**
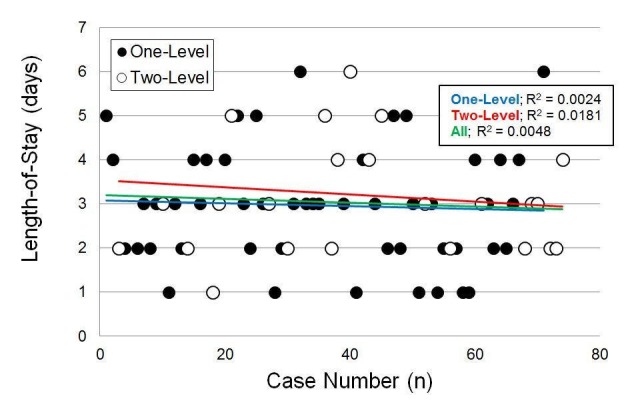
Patient length-of-stay vs. case number; case number was not a significant predictor (p = 0.51)

Patient hospitalization LOS time did not significantly differ by age, sex, the number of surgical levels, or BMI (p > 0.99) (Tables 3-4). When modeling LOS as a function of the case number, the most likely change point was after 40 cases. However, in the multiple linear regression model, the case number was not a significant predictor of LOS (p = 0.51) and there was no significant difference between patients' LOS before and after the 40th case (p = 0.36) (Figure 5).

### Complications

Intraoperatively, one patient (Case #19), undergoing L4-S1 fusion, suffered a spinous process fracture at L5-S1, resulting in intraoperative unilateral PSF at L5-S1. No other intraoperative ISPF device-related complications were observed. Nine patients suffered intraoperative durotomies, all of which were resolved with the application of fibrin glue (Case: #2, 28, 32, 35, 43, 55, 62, and 71).

Mean follow-up was 13.3 months. Two patients suffered from perioperative infections, both of which resolved with conservative treatment consisting of oral antibiotics (Case # 24, 65). Six patients (8.2%) required surgical revision due to observed interbody cage migration (mean time to migration event: 7.5 weeks). Two migration cases (due to cage under-sizing) required removal of the ISPF device during interbody revision, followed by bilateral PSF, while the other four interbody revisions did not require modification of the ISPF device. In all cases of cage migration, the ISPF device(s) were noted to be intact and stable. No complications due to unprovoked ISPF device migration and/or failure were observed postoperatively.

## Discussion

While bilateral PSF remains the most readily accepted means to supplemental stability in MIS TLIF, procedural adoption often requires overcoming a significant learning curve [1-5]. Accordingly, there has been a continued emergence of alternative or hybrid MIS TLIF techniques in which less fixation and/or less disruptive fixation are leveraged in an effort to reduce operative demand and subsequently marginalize any associated learning curve. However, despite the perceived benefits of these surrogate techniques, little effort has been made in the literature to formally substantiate whether or not they may possess their own inherent learning curve. The objective of this study was to assess whether an intraoperative learning curve may exist for TLIF + ISPF, a less commonly practiced alternative with the TLIF platform.

In considering EBL, LOS, and fluoroscopy time, it was found that the number of preceding cases was not an indicator of outcome improvement, but rather that outcomes were consistent throughout. While these trends are inherently specific to this surgeon author team alone, they support the proposed ideal that the favorable anatomical proximity of ISPF may help mitigate any intraoperative learning curve.

These trends are contrasting to several reports of MIS TLIF + BPSF in which a learning curve has been observed. Nandyala, et al. found in their first 65 primary one-level cases that there were significant differences in EBL, use of intraoperative fluids, and duration of anesthesia between their early group of 33 patients and the latter group of 32 patients [4]. Lee, et al., evaluating their first 90 patient series, found that mean fluoroscopy time and usage of patient-controlled analgesia differed significantly between their early group of 44 patients and the latter group of 46 patients [3]. Interestingly, EBL, time to ambulation, and LOS were not significantly different between the groups. Lastly, Lee, et al., when assessing their first 60 one-level cases, found that significant differences in EBL and time to ambulation existed for their early group of 22 patients and their latter group of 38 patients [2].

While the intraoperative outcomes demonstrated in this study indicate that TLIF + ISPF may be executed without a significant procedural learning curve, it is also critical to consider how the raw outcomes compare to those of traditional MIS TLIF + BPSF. It would be incorrect to assert that the lack of a learning curve indicates a procedure as inherently advantageous. If the outcomes are not comparable or favorable to those of established techniques then any perceived ease of adaptability is a moot point.

In considering studies evaluating one-level MIS TLIF + BPSF, intraoperative EBL, LOS, and fluoroscopy time have been shown to range from 55 ml to 410.6 ml, 2.3 to 14.6 days, and 43 to 105.5 seconds, respectively [15, 16-19]. Single-level fusion patients in the current study demonstrated comparable/favorable mean values for EBL (184.8 ml), LOS (2.9 days), and fluoroscopy time (31.4 sec), respectively. Given the established challenges of placing PSF, as well as the increased number of individual hardware components, decreased use of fluoroscopy time with ISPF is not unanticipated. Additionally, as is the case with any TLIF technique, the delineation between those intraoperative outcomes associated specifically with the interbody placement and those associated with posterior fixation is not readily possible and must be given fair consideration when assessing these metrics.

Studies specifically evaluating two-level MIS TLIF + BPSF have demonstrated EBL and LOS to range from 124 to 481.2 ml and 5.1 to 9.3 days, respectively, while one study reported mean fluoroscopy time at 45 seconds [17, 20-23]. In this case series, two-level patients demonstrated comparable EBL values (394 ml), while LOS (3.2 days) and fluoroscopy time (42.1 sec) values appeared favorable to those of MIS TLIF + BPSF.

Consideration of MIS TLIF application in difficult-to-treat populations must also be given, particularly in elderly and obese cohorts. When evaluating MIS TLIF + PSF in Class I-III obese patients, Lau, et al. found mean EBL and LOS values to range from 141.7 to 269.6 ml and 3.0 to 3.6 days, respectively [24]. In this study, mean values for EBL, LOS, and fluoroscopy in obese patients, including all obesity classifications and both one- and two-level fusions, were 312 ml, 3.0 days, and 38.9 seconds, respectively. These values did not significantly differ from those of non-obese patients (p ≥ 0.10).

With respect to patient age, several prior studies have assessed the intraoperative outcomes of elderly cohorts undergoing MIS TLIF + PSF. Wu, et al. found mean EBL and LOS to be greater for elderly patients undergoing bilateral PSF in one- and two-level cases, although only LOS reached significance (160.1 vs. 149.9 ml, 5.1 vs. 4.5 days) [25]. Similarly, Lee, et al. found mean EBL and LOS to be greater for elderly patients undergoing one-level fusion, although results did not reach significance (100 vs. 93.4 ml, 3.89 vs. 2.49 days) [26]. In the current study, elderly patients (≥ 60 years) demonstrated mean EBL (266.7 ml), LOS (3.4 days), and fluoroscopy time (39.4 sec) values that were comparable to those reported in the literature for PSF and not significantly differing from non-elderly subjects (p ≥ 0.06).

Perioperative complications and subsequent revisions were present in this study, particularly involving IB cage migration. All cases of cage migration (n = 6, 8.2%) were observed in the latter half of the case series (≥Case #34). Given the many factors that can contribute to cage migration, including, but not limited to, cage size, shape, the number of fused segments, endplate shape, disc height, and bone mineral density, it is difficult to identify a definitive root cause [27]. A most recent meta-analysis of comparative MIS vs. Open TLIF studies by Khan, et al. reported cage migration rates of 0-5.6% and 0-8.3% for MIS and Open, respectively [28].

While the cage migration incidence rate observed in this study is within the upper limits reported in the literature, it does create just cause for further consideration. Given that ISPF functions via an off-set from the anterior column, careful attention to the moment arm created during implantation is critical in ensuring appropriate compression of the IB cage. Excessive compression or distraction of the spinous processes with an ISPF device can result in inadvertent loading/unloading of the cage, predisposing it to migration and/or subsidence.

In additional to cage migration complication, 2.7% (n = 2) and 12.2% (n = 9) of subjects suffered perioperative infections and/or incidental durotomies, respectively. Similar MIS TLIF learning curve studies with PSF have expressed superficial or deep wound infections in 0 to 9.4% of subjects and rates of incidental dural tears, lesions, durotomies, and/or subsequent cerebrospinal fluid leaks in 0 to 12.5% of subjects, respectively [21, 29]. However, it should be noted that such complications are not necessarily inherent to the posterior fixation aspect of the TLIF technique and should largely be considered a potential limitation of the collective procedure.

### Study limitations

This study was performed retrospectively with intrinsic limitations such as patient selection bias. The assumption was also made that any intraoperative learning curve would be captured within the first 74 cases. It is possible that changes in perioperative outcomes could be seen with a larger series size. However, a volume of 74 cases falls within the range of previous MIS TLIF + BPSF learning curve reports (60 to 90 cases) [2-4]. Furthermore, all of the aforementioned reports were also performed retrospectively without a control cohort [2-4].

This study also evaluated perioperative outcomes with respect to the cumulative surgical procedure, including both the IB and posterior fixation approaches. Stratification of outcomes specific to the interbody approach and posterior fixation alone may provide a more focused analysis as to how intraoperative metrics function relative to specific aspects of the surgery.

Furthermore, a general limitation of any learning curve analysis is the inability to fully characterize prior experiences that may impact surgeon adaptability. In the case of this study, previous surgical experience in the lumbar spine, regardless of hardware application, makes any subsequent techniques more intuitive given a baseline of familiarity. Therefore, it is important to evaluate a surgeon learning curve across a spectrum of surgical teams with both varied experience levels and specializations.

## Conclusions

This study analysis served as the first report in the literature to assess whether minimally disruptive TLIF + ISPF may possess an intraoperative learning curve. In considering both one- and two-level cases, outcome trends demonstrated that EBL, LOS, and fluoroscopy time were not associated with the number of preceding cases. Furthermore, mean outcome values for these metrics were comparable and/or favorable to those reported in the literature for MIS TLIF + BPSF, as well as demonstrating no significant relationship with elderly and obese subjects. While no ISPF device failures or migrations were observed, the notable number of cage migrations warrants further consideration as to the biomechanical mechanisms of ISPF in supporting TLIF.

This study supports the notion that TLIF + ISPF can be a readily adopted procedure, performing well across a wide spectrum of demographics. However, despite these advantageous trends, the authors emphasize that further assessment of long-term healing outcomes is essential in fully characterizing both the efficacy and the indication learning curve for the TLIF + ISPF technique.
